# The Utility of Serological Analysis for *Neospora caninum* Infection in Dairy Cattle Farms Management: Serological Investigation and Evaluation of the Effects on Reproductive and Productive Performances in Two Study Herds in Northern Italy

**DOI:** 10.3390/ani12060786

**Published:** 2022-03-20

**Authors:** Luca Villa, Alessia Libera Gazzonis, Emanuele Fumagalli, Sergio Aurelio Zanzani, Maria Teresa Manfredi

**Affiliations:** 1 Department of Veterinary Medicine and Animal Sciences, Università degli Studi di Milano, Via dell’Università 6, 26900 Lodi, Italy; alessia.gazzonis@unimi.it (A.L.G.); sergio.zanzani@unimi.it (S.A.Z.); mariateresa.manfredi@unimi.it (M.T.M.); 2 Independent Researcher, 24121 Bergamo, Italy; fumagalli.emanuele@yahoo.it

**Keywords:** neosporosis, dairy cows, herd efficiency, serology, abortion

## Abstract

**Simple Summary:**

Among infectious agents triggering reproductive disorders in cattle, *Neospora caninum*, an obligate intracellular protozoan parasite, is a major cause of abortion in cows worldwide. A serosurvey was designed in two cattle herds, both located in northern Italy and with similar reproductive management features, recruited as a case study. The clinical outcome and the effects on herd performances were evaluated in association with the seropositivity to *N. caninum*. This study showed that the integration of serological analysis results for *N. caninum*, the reproductive and productive parameters, and the information on herd performances, could give indications for the application of control strategies.

**Abstract:**

*Neospora caninum* is recognized as a major cause of abortion in cattle, determining economic losses, particularly in dairy industries. To evaluate the impact of neosporosis on herd efficiency, and to understand how the serological analysis for *N. caninum* is explicative of the farm picture, an epidemiological study was designed in two dairy farms recruited as a case study. Blood samples were collected from 540 animals, including cows and heifers over 12 months, and analyzed by an indirect immunofluorescent antibody test with subsequent antibody titration. Overall, 94 animals (17.4%) were identified as positive to *N. caninum* antibodies (15.5% and 18.5% in Farm 1 and Farm 2), with differences between the farms concerning the antibody titers (Chi-square, *p*-value = 0.04), particularly in cows (Chi-square, *p*-value = 0.018). Consequently, a different pattern of abortion episodes was depicted in the two investigated farms. Considering reproductive and productive performances, the number of inseminations necessary to make an animal pregnant was higher in seropositive cows (2.4 and 2.9 in Farm 1 and 2, respectively) than in seronegative ones (2.1 and 2.4 in Farm 1 and 2, respectively). Similarly, particularly in Farm 1, the number of days in milk of not-pregnant cows was higher in seropositive (167.7) than seronegative animals (133.4). Moreover, although the association between *N. caninum* infection and milk production is still unclear, both the daily milk production and the 305-mature equivalent milk yield were lower in seropositive (31.02 and 11,838.94) than seronegative cows (33.59 and 12,274.88) in Farm 1; an opposite pattern was otherwise depicted in Farm 2. The study showed that even if *N. caninum* circulated equally in the two herds, the dynamics of the parasite infection and its outcome may be different, also depending on the specific situation of the farm. In this way, the integration of serological analysis for *N. caninum*, the reproductive and productive parameters, and the information on herd performances, could give specific indications for the application of control strategies.

## 1. Introduction

The dairy industry is a relevant economic sector in Italy with 25,525 farms hosting 2,642,918 animals and producing 12,092,861 tons of milk yearly with a turnover of about €16.3 billion. More than 80% of the milk is produced in dairy farms in northern regions, particularly in the Po Valley in Lombardy. This territory stands out for the high density of cattle farms, mainly based on Italian Holstein Friesian under the intensive production system. According to the Italian National Zootechnical Registry, Lombardy accounts for 20.77% and 41.23% of all farms and animals in Italy, with an annual production of 5,318,112 tons of milk (https://report.assolatte.it/2019/Appendice_Statistica_Rapporto2019.pdf; last access on 13 February 2022).

The dairy cattle production industry depends on herd reproductive efficiency, which has a major impact on economic success. Therefore, the optimization of reproductive performance also passes through the control of reproductive diseases contributing not only to the maintenance of health and welfare of dairy cows but also to the economic success of the farm [1].

Among infectious agents triggering reproductive disorders in cattle, *Neospora caninum*, an obligate intracellular protozoan parasite, is a major cause of abortion in cows worldwide [2]. Domestic dogs and wild canids (gray wolves, coyotes, and dingoes) are the definitive hosts shedding unsporulated oocysts in their feces; various species were reported as intermediate hosts of the protozoan, including ruminants [2]. In cattle, *N. caninum* can be transmitted horizontally by ingestion of food or water contaminated by sporulated oocysts. Otherwise, it can be transmitted vertically, i.e., through transplacental congenital transmission from an infected dam to her fetus during pregnancy. Vertical transmission can be classified as exogenous transplacental transmission if it occurs because of oocyst-derived infection in a pregnant dam, or as endogenous transplacental transmission in the case of the reactivation of the parasite in a persistently infected dam during pregnancy. It should be considered that recrudescence of latent or persistent infection in the dam during gestation is the main cause responsible for the maintenance of the parasite infection in endemically infected dairy cattle herds [3]. *N. caninum* is one of the most efficiently trans placentally transmitted organisms in cattle: indeed, up to 95% of calves are born infected [3]. The outcome of infection in pregnant cows can be the abortion of the fetus, or the birth of a still-born calf, a calf with neurological clinical signs, or a clinically healthy persistently infected calf [4]. Abortion is the main clinical manifestation of *N. caninum* infection or bovine neosporosis: this event may have an epidemic character when the abortion appears as a temporary outbreak with 10–15% of cows at risk of aborting within 4–8 weeks. In contrast, an abortion problem is regarded as endemic if it persists in the herd for several months or years, being related to endogenous transplacental transmission [5]. However, intermediate abortion patterns (mixed patterns), possibly caused by both endogenous and exogenous transmission, can also be observed in the field. Cows of any age may abort from 3 months of gestation to term, with most abortions occurring at 5–7 months of gestation [3]. Moreover, the parasite was also associated with other reproductive problems, i.e., stillbirth and perinatal mortality [6]. Besides, a few reports suggested an adverse effect of *N. caninum* in early pregnancy [7,8,9] manifested as a return to service, increased time to conception or infertility, and increased calving intervals in seropositive cows [10,11]. Furthermore, some studies also reported a reduction in milk yield in dairy cattle [12,13,14,15] and reduced weight gain and feed efficiency in beef calves and steers [16,17], even if these data are still controversial [11]. The major economic impact of neosporosis is due to reproductive failure in dairy cows; indeed, fetal death represents direct costs, even if other indirect losses in the farm should also be considered, such as expenses for professional help and diagnosis and lengthened intervals for rebreeding and replacement of culled cows [18,19,20]. To date, no safe and effective chemotherapy for bovine neosporosis, and no vaccine against abortion or *N. caninum* transmission, are available. For the control of bovine neosporosis, regional differences in its epidemiology should be considered; besides, control programs should incorporate a cost–benefit calculation to compare the expenses of testing and control measures with the benefit of reduced economic losses due to *N. caninum* infection or abortion. In *N. caninum*-free herds, prevention through standard biosecurity measures is the primary goal; instead, in *N. caninum*-infected herds, control measures include the use of alternatives in the reproductive management of the herd but there are also “Test and cull” and “doing nothing” strategies [3]. *N. caninum* infection in cattle was reported from most parts of the world with serological prevalence values varying among and within countries, between the regions, and between beef and dairy cattle [11]. A pooled prevalence of 13% was evidenced in Europe [21]. In Italy, only a few epidemiological studies were conducted on cattle with seroprevalence values varying from 44.1% to 77.8% at the herd level according to geographical regions and analytical methods [22,23,24]. Moreover, a recent study on bovine aborted fetuses reported a molecular prevalence of *N. caninum* of 27.8% in the Lombardy region, confirming the involvement of the parasite in reproductive failure in dairy cattle farms in northern Italy [25].

In clinical practice, neosporosis is included among differential diagnoses when the abortion rate within the herd exceeds the normality range [26]. In general, serological screening for *N. caninum* antibodies on some animals or the whole herd, also due to the low costs, is carried out; however, serological data may not be correctly interpreted considering the whole clinical picture of the farm. Indeed, serological tests are sometimes used before or in substitution of the etiological diagnosis on aborted fetuses, often directly removed by the breeder or the farmworkers, thus making the direct diagnosis not feasible. Moreover, to depict the reproductive status of cows, clinical examination (particularly gynecological) and laboratory analyses, with also the evaluation of managerial factors affecting the health and well-being of animals, should be included. Therefore, a serosurvey was designed aimed at exploring the seropositivity of *N. caninum* and its competence to highlight the reproductive and productive reduced efficiency in cattle herds affected by neosporosis. In this respect, two cattle herds, both located in northern Italy and with similar reproductive management features, were recruited as a case study: the clinical outcome and the effects on herd performances were evaluated in association with the seropositivity to *N. caninum*.

## 2. Materials and Methods

### 2.1. Farms’ and Area Description

Two dairy cattle farms with similar management were selected. Farm 1 and Farm 2 hosted 360 (170 lactating cows, 20 dry cows, and 170 heifers) and 530 animals (240 lactating cows, 40 dry cows, and 250 heifers), with a mean daily production at the time of sampling of 37 and 36.5 liters of milk, respectively. In both farms, a reduction in herd performances, in terms of reproductive or productive parameters, was evidenced. Both farms are family-run and grow their crops to feed the cattle. Besides, both Farm 1 and Farm 2 performed embryo transfer for genetic improvement of Holstein Friesian cattle. Previous analyses evidenced that the selected farms reported cases of abortion due to *N. caninum*; however, no specific control plan for neosporosis was implemented. Both herds used the AfiFarm Software (AfiMilk Ltd., Kibbutz Afikim, Israel) for farm management.

The farms were located in northern Italy, in the provinces of Lodi (45°21′17″ N 9°22′31″ E) and Bergamo (45°38′23″ N 9°30′23″ E), respectively. Both sites belong geographically to the “Bassa Padana” in the Po valley and have an average altitude of about 110 m above sea level. The climate is continental with a hot muggy summer and cold and foggy winter.

### 2.2. Sample and Data Collection

All lactating and dry cows and heifers above 12 months of age present in the two farms at the time of sampling were included in the study (*n* = 540). During the blood sampling campaign for prophylaxis of infectious diseases planned at both farms in April 2018, a fraction of the collected blood sample was preserved aimed at the detection of anti-*Neospora caninum* antibodies. Blood samples were collected in tubes without anticoagulants by puncturing the tail vein using a Vacutainer^®^ sterile collection system and preserved refrigerated during transportation to the laboratory within a few hours. Once in the laboratory, sera were separated by centrifugation (2120× *g*, 15 min) and stored at −20 °C until serological analysis.

Individual data and information regarding reproductive and productive parameters of cattle were collected by the farm managerial software. Individual data including breed, sex, age, productive category, number of lactations (only for cows), and origin of the animals were collected. Concerning reproductive parameters, data on episodes of embryonal reabsorption and abortion in the period between January 2017 and April 2018, the number of inseminations, and days in milk were recorded. Regarding productive parameters, daily milk production (kg of milk per day) and 305-mature equivalent milk yield, were noted: the latter parameter adjusts all cows to the same age, season of calving and lactation length, and to the geographic area of the herd (Si@llEvA, Italian Breeder Association, www.sialleva.it; last access on 13 February 2022).

Overall, 540 animals, 340 and 200 from Farm 1 and 2, respectively, were included in the study. All sampled animals were female Holstein Friesian cattle and all of them were born in the farm. Both heifers and cows (both lactating and dry) were considered. The mean age of the sampled cattle was 42.3 months (SD = 20.4, Min–Max = 9.8–115.4): in particular, cattle had a mean age of 39.8 (SD = 14.5, Min–Max = 22.0–110.1) and 43.1 months (SD = 21.8, Min–Max = 9.8–115.4) in Farm 1 and 2, respectively.

### 2.3. Serology

Serum samples were analyzed for the detection of anti-*N. caninum* specific antibodies by using a commercial immunofluorescence antibody test (MegaScreen^®^ FLUO NEOSPORA caninum, Megacor, Hoerbranz, Austria). The procedure was carried out according to the manufacturer’s instruction, as previously described [27]. An initial dilution of 1:160 was used for screening; subsequently, seropositive samples were twofold serially diluted for the determination of the end-point antibody titer.

### 2.4. Data Analysis

Seroprevalence of *N. caninum* was calculated considering the two farms and the productive categories (heifers and cows) of sampled animals. A chi-square test was used to infer the difference in *N. caninum* seroprevalence considering the two farms, the productive category, the age (<2 years, 2–4 years, >4 years), and the number of lactations (1, 2, 3, 4 and more); similarly, differences in antibody titers considering the same variables were explored using a Chi-square test. The effects of the parasite infection on the herd performances in the two farms were also investigated through univariate generalized linear models (GLMs) with linear distribution. Data concerning each farm were singularly considered. Reproductive (number of inseminations, days in milk) and productive parameters (daily kg of milk, 305-mature equivalent milk yield) were separately considered and entered in each model as dependent variables. The binary outcome (presence/absence of anti-*N. caninum* antibodies) based on serology results was used as the independent variable. The level of significance was set at *p*-values of <0.05. Statistical analysis was performed using SPSS software (Statistical Package for Social Science, IBM SPSS Statistics for Windows, version 25.0., Chicago, IL, USA).

## 3. Results

Overall, 94 animals scored positive for *N. caninum* antibodies with a prevalence of 17.4%. Considering the two study farms, similar seroprevalence values were evidenced, with a slightly higher value in Farm 2 (18.5%) than in Farm 1 (15.5%). In both farms, the presence of *N. caninum* antibodies was higher in cows than in heifers. However, data analysis revealed that no statistically significant difference was evidenced in the seroprevalence of infection between the two farms and between the production categories (Chi-square, *p*-value > 0.05). Detailed serological results and antibody titers of *N. caninum* infection in the sampled farms, according to both immunofluorescence antibody test results and the considered categories of animals, are reported in Table 1.

Concerning the seropositivity in relation to age and the number of lactations, the seroprevalence was higher in older animals and cows with four and more lactations (Table 2). No statistically significant difference was evidenced in the seroprevalence of *N. caninum* infection considering both the age and the number of lactations of sampled animals (Chi-square, *p*-value > 0.05).

Moreover, the distribution of antibody titers was found to be different between the two study farms, with higher titers in Farm 1 than in Farm 2: indeed, 29.0% and 6.0% of seropositive animals showed an antibody titer higher than 1:640 titer in Farm 1 and Farm 2, respectively. A statistically significant difference was underlined in the distribution of antibody titers between the two study farms, considering both the total number of animals (Chi-square, *p*-value = 0.04) and also the cows (Chi-square, *p*-value = 0.018), whereas there was no significant difference between only the heifers (Chi-square, *p*-value > 0.05).

Regarding the episodes of abortions, a different pattern was depicted in the two investigated farms. Between January 2017 and April 2018, a total of 40 abortions were recorded, 14 and 26 in Farm 1 and Farm 2, respectively. Abortions occurred in the first (27), second (11), and third (2) trimester of pregnancy. Nine abortions from seropositive cows were recorded (Farm 1: 4; Farm 2: 5) corresponding to 22.5% of all recorded abortions. In Farm 1, the rate of abortion in seropositive cows was 19%; all these abortions (4) occurred in the second trimester of gestation and showed high antibody titers (1:320 and 1:640). Instead, in Farm 2, the abortion rate in seropositive cows was 9.8%, with two cows aborting in the first trimester showing cut-off antibody titer, while the three cows that aborted in the second trimester evidenced antibody titers of 1:160 and 1:320. (Table 3).

Finally, reproductive and productive parameters were considered to investigate the impact of *N. caninum* infection in the two farms. In both farms the number of inseminations necessary to make an animal pregnant was higher in seropositive animals than in seronegative animals (2.1 vs. 2.4 in Farm 1 and 2.4 vs. 2.9 in Farm 2); moreover, insemination mean was higher in Farm 2 than in Farm 1. The number of days in milk of not-pregnant cows was higher in seropositive cows both in Farm 1 and in Farm 2. However, these values were particularly high in relation to the reference interval in all cows in Farm 2. Concerning productive parameters, both the daily production and the mature equivalent milk yield were lower in seropositive than seronegative cows in Farm 1; instead, in Farm 2 both parameters were higher in seropositive animals if compared to seronegative ones (Table 4). Statistical analysis by GLMs did not show any association between *N. caninum* infection and the considered reproductive and productive variables.

## 4. Discussion

In the study, a serological screening on *N. caninum* was performed in two dairy cattle farms in the Lombardy region (northern Italy) aimed at evaluating the impact of the infection on herd efficiency and to understand if serology for *N. caninum* could be explicative of reproductive and productive problems in these farms. The results showed that the parasite circulates with similar intra-herd prevalence values in both farms (15.5% and 18.5% in Farm 1 and 2, respectively); however, the impact of neosporosis on the reproductive and productive efficiency of the study herds appeared different.

The prevalence values were in accordance with both other Italian [22,23] and international studies [11,21]. However, these were lower than prevalence data from other Italian farms [28] and if compared to studies demonstrating a diffuse exposure to *N. caninum* in domestic and wild species in the study area [27,29,30,31].

No differences were found in the seroprevalence values neither between the two farms nor considering the production categories, age, and the number of lactations; however, *N. caninum* seropositivity was higher in cows than in heifers in both farms (Table 1 and Table 2). Indeed, as previously demonstrated, the risk of testing seropositive to *N. caninum* may increase with the age, parity number, number of pregnancies, gestation number, or number of lactations [32].

A statistically significant difference in the distribution of antibody titers between the two farms, considering both the total number of animals and the cows only, was evidenced; indeed, in Farm 1 antibody titers results were higher compared to Farm 2 (Table 1). Since it was demonstrated that seropositive cows are more likely to abort than seronegative cows and the risk of abortion increases to the increase in levels of specific anti-*N. caninum* antibodies [33,34], the risk for abortion due to *N. caninum* seems to be higher for cows in Farm 1. Indeed, a high level of antibodies could be indicative of high dose infection and/or efficient multiplication of the parasite in the infected host regarding post-natal infection (exogenous transplacental transmission); instead, in the case of latent infection (endogenous transplacental transmission), a high level of antibodies could reflect the presence or intensity of recurrence of a current chronic infection. In this regard, it was reported that the intensity and the duration of the increase in anti-*N. caninum* specific antibodies during gestation could be related to the risk of fetal infection [35,36,37].

As a result of the difference in the distribution of antibody titers, a peculiar abortion pattern was also evidenced in each of the two study farms (Table 3). Indeed, in Farm 1, the rate of abortion was higher in seropositive than in seronegative cows. In this herd, abortion episodes mostly occurred in the second trimester, the typical gestational period for *N. caninum* induced abortion; besides, seropositive aborting cows showed high antibody titers, suggesting a possible involvement of the parasite in the pathogenesis. As previously reported, in the absence of a specific control program, the percentage of abortions in seropositive cows is greater than in the seronegative ones [28]. Instead, in Farm 2, the rate of abortion was very similar between seropositive and seronegative cows. Here, embryonic death and resorption episodes prevailed, with the evidence of repeat breeders’ cows: indeed, most of the abortions were concentrated in the first trimester of gestation, and this time frame is not suggestive of *N. caninum* infection, even if some studies indicated an adverse effect of the parasite in early pregnancy [7,8,9]. Moreover, only a few abortions episodes were evidenced in the second trimester in seropositive cows with low antibody titers: this pattern is less suggestive of an involvement of *N. caninum* in reproductive failure episodes, suggesting the occurrence of other infectious or not infectious (e.g., managerial, nutritional) causes in the lack of reproductive efficiency of the herd.

Regarding reproductive performances, even if no statistically significant difference was evidenced, in both farms the mean number of inseminations was higher in seropositive cows especially in those from Farm 2 (Table 4). These results agree with some previous reports that also demonstrated an adverse effect of *N. caninum* on reproductive efficiency in dairy herds [38]. In particular, the number of inseminations for conception was higher in seropositive animals than in seronegative ones [7,8,39,40]. Although a few studies did not observe any influence of *N. caninum* infection in early pregnancy [33,41], it was suggested that an increase in the number of services necessary for cows to conceive may indicate embryonic death followed by fetal reabsorption, leading to a return to heat [9,42]. Moreover, some studies also reported an increased calving to conception interval in seropositive cows [9,39,40].

Consequently, productive parameters may also be influenced due to reproductive failure. In this regard, a different pattern was evidenced in the two study herds, even if no statistically significant difference was evidenced. Indeed, both the daily milk production and the mature equivalent milk yield was lower in seropositive cows in Farm 1. Instead, only a slight difference in productive performances was evidenced between seronegative and seropositive cows in Farm 2, with higher daily milk production and mature equivalent milk yield in seropositive ones (Table 4). In this regard, data from previous studies report a decrease or an increase in milk production in infected cows [12,13,14,43] or no effect of the parasite on milk yield [8,44]. Particularly, a study from Gonzalez-Warleta et al. [45] evidenced that milk production losses mainly occur in highly positive farms. According to Tiwari et al. [15], the reduction in milk production in seropositive animals may be related to reduced productivity after an abortion in first-lactation heifers, possibly related to abortion sequelae, since any infection might leave lesions or cost energy to the cow [46]. It was also recently suspected that other protozoan diseases may be implicated in the reduction in milk production in cattle [47]. It should be considered that since milk production depends on many factors, i.e., the farm effect, as the sum of genetics, management, and environmental effects, it is difficult to assess if neosporosis could have a substantial effect on productive parameters [14].

Even if the circulation of the neosporosis was comparable in terms of seroprevalence values between the two farms, the dynamics of the parasite infection and its outcome was peculiar, also depending on the specific situation of the farms. In this way, the integration of serological analysis results for *N. caninum*, the reproductive and productive parameters, and the information on herd performances, could give indications for the application of control strategies.

Indeed, concerning Farm 1, the evidence of higher antibody titers for *N. caninum*, particularly in cows, and the record of abortions in the second trimester in seropositive cows with high antibody titer, with only a mild effect on reproductive and productive parameters, seems to suggest that in this herd *N. caninum* infection may be endemic, leading to problems for the herd economy. Therefore, the application of a monitoring program for neosporosis, including regular serological screening of the animals and diagnosis on aborted fetuses, may be indicated.

Instead, in Farm 2, the main problem seems to be linked to early fetal death with the evidence of “repeat breeders”. The minor percentage of abortion episodes recorded in the second trimester was evidenced in seropositive cows with low antibody titers. Besides, the number of inseminations were higher both in seropositive and seronegative cows, days in milk were generally very high in all animals, whereas no alterations were detected in productive parameters. Hence, in this farm, other infectious or managerial causes should also be investigated.

Among variables contributing to the differences in the two study farms, the intensity of horizontal transmission, the circulation of other abortigenic pathogens, and all those managerial features (i.e., feeding, lame management, milking system) that could affect animal health and welfare, should also be considered.

The serological screening of the herd is a valid method for estimating the prevalence of *N. caninum* at both individual and farm levels; in addition, the evaluation of the serological status of cattle using IFAT also allows the antibody titration in seropositive animals. The information regarding the antibody titer of the cows could be a predictive tool to identify cows at increased risk of abortion in infected farms [48]. Thus, the determination of the antibody titer in the study farms could have an important practical application considering both widely used embryo transfers: indeed, the antibody titer could help the breeder in the choice of the right animal to implant the embryo. In this way, in both study farms, the use of embryo transfer, already routinely applied, may be useful in the control of neosporosis. Among the other control strategies for neosporosis applicable to these farms, the use of “Test and cull”, i.e., the culling of infected cows, is an effective control option, but not always economically viable; besides, in Farm 1, artificial insemination of seropositive cows with semen from beef bulls could have a beneficial effect. Finally, in both farms, the implementation of biosecurity measures should always be considered. Moreover, as previously suggested and consolidated for *T. gondii* infection in ruminants [49,50], antibody detection in individual and bulk tank milk could also represent a valid alternative to serological analysis for *N. caninum* in cattle, to estimate and monitor parasite herd prevalence [45,51,52].

## 5. Conclusions

In conclusion, the study showed that although *N. caninum* circulates equally in farms, the dynamics of the parasite infection and its outcome were different. Serological screening by IFAT proved to be a useful diagnostic tool to determine not only herd seroprevalence, but also to identify, through the determination of antibody titer, animals at higher risk of abortion.

Although further studies to be conducted on more animals from different farms are needed to clarify the role of *N. caninum* in alterations of reproductive and productive parameters in cattle, the preliminary data obtained in these two case-study farms may suggest an impact of the parasite on herd performances. Finally, the integration of serological analysis results for *N. caninum*, the reproductive and productive parameters, and the information on herd performances, could give indications for the application of control strategies.

## Figures and Tables

**Table 1:** Serological prevalence of *Neospora caninum* infection in cattle from two farms in northern Italy according to both immunofluorescence antibody test and productive categories of cattle. Antibody titers are also reported.

Farm	Category	% (Positive/Examined)	CI 95%	Antibody Titer			
				1:160 % (*n*)	1:320 % (*n*)	1:640 % (*n*)	>1:1280 % (*n*)
Farm 1	Cows	19.1 (21/110)	11.7–26.4	23.8% (5)	28.6% (6)	33.3% (7)	14.3% (3)
	Heifers	11.1 (10/90)	4.6–19.6	30.0% (3)	50.0% (5)	20.0% (2)	0.0% (0)
	Total	15.5 (31/200)	10.5–20.5	25.8% (8)	35.5% (11)	29% (9)	9.7% (3)
Farm 2	Cows	19.7 (51/258)	14.9–24.6	37.2% (19)	47.0% (24)	5.8% (3)	9.8% (5)
	Heifers	14.6 (12/82)	6.9–22.3	50.0% (6)	41.7% (5)	8.3% (1)	0.0% (0)
	Total	18.5 (63/340)	14.4–22.7	39.7% (25)	46.0% (29)	6.3% (4)	7.9% (5)
Total	Cows	19.6 (72/368)	15.5–23.6	33.3% (24)	41.7% (30)	13.9% (10)	1.1% (8)
	Heifers	12.8 (22/172)	7.8–17.8	40.9% (9)	45.5% (10)	13.6% (3)	0.0% (0)
	Total	17.4 (94/540)	14.2–20.6	35.1% (33)	42.5% (40)	13.8% (13)	8.5% (8)
CI 95% = confidence interval 95%							

**Table 2:** Serological prevalence of *Neospora caninum* infection in cattle from two farms in northern Italy according to both age and number of lactations.

		Farm 1 % (Positive/Examined)	Farm 2 % (Positive/Examined)
**Age**	**<2 years**	11.9% (11/92)	17.1% (12/70)
	2–4 years	15.5% (13/84)	15.9% (24/151)
	>4 years	29.1% (7/24)	22.7% (27/119)
**Number of lactations**	**1**	17.7% (11/62)	19.8% (19/96)
	2	20.0% (7/35)	16.0 (12/75)
	3	14.3% (1/7)	25.5% (12/47)
	4 and more	33.3% (2/6)	23.1% (9/39)

**Table 3:** Episodes of abortion occurring in the study farms between January 2017 and April 2018. Antibody titer of seropositive aborting cows is also reported.

Farm	Trimester of Abortion	Abortions from *N. caninum* Positive Cows	Total Abortions	Antibody Titer
1	I	0	6	-
	II	4	8	1:320, 1:640
	III	0	0	-
2	I	2	21	1:160
	II	3	3	1:160, 1:320
	III	0	2	-

**Table 4:** Reproductive and productive performances in seronegative vs. *N. caninum* seropositive cows from two cattle farms in northern Italy.

	Farm 1		Farm 2	
	Seronegative (Mean ± SD)	Seropositive (Mean ± SD)	Seronegative (Mean ± SD)	Seropositive (Mean ± SD)
**Number of inseminations**	2.1 ± 1.5	2.4 ± 1.4	2.4 ± 1.9	2.9 ± 1.6
**Days in milk (DIM)**	133.4 ± 42	167.7 ± 54.3	219.6 ± 94	221.6 ± 117.7
**Daily milk production (Kg)**	33.59 ± 8.27	31.02 ± 4.99	39.92 ± 7.68	40.37 ± 8.03
**305-Mature Equivalent Milk Yield**	12,274.88 ± 2434.806	11,838.94 ± 2337.747	12,144.59 ± 1851.022	12,464.93 ± 2029.262

## Data Availability

The datasets used and/or analyzed during the current study are available from the corresponding author on reasonable request.
